# Study of Children Aged Under 2 Years Admitted With RSV at Four Australian Hospitals [2021–2022]

**DOI:** 10.1111/jpc.16769

**Published:** 2025-01-18

**Authors:** Nigel W. Crawford, Annette Alafaci, Julia E. Clark, Joshua R. Francis, Christopher C. Blyth, Catherine Pienaar, Cara Minney‐Smith, Sonia Dougherty, Anjalee Panditha, Laura Francis, Ian G. Barr

**Affiliations:** ^1^ The Royal Children's Hospital Parkville Victoria Australia; ^2^ Surveillance of Adverse Events Following Vaccination in the Community (SAEFVIC), Murdoch Children's Research Institute Parkville Victoria Australia; ^3^ Department Paediatrics University of Melbourne Parkville Victoria Australia; ^4^ Infection Management Service, Childrens Health Queensland Brisbane Queensland Australia; ^5^ University of Queensland Brisbane Queensland Australia; ^6^ Global and Tropical Health Division, Menzies School of Health Research, Charles Darwin University Darwin Northern Territory Australia; ^7^ Department of Paediatrics Royal Darwin Hospital Darwin Northern Territory Australia; ^8^ Wesfarmers Centre of Vaccines and Infectious Diseases, Telethon Kids Institute Nedlands Western Australia Australia; ^9^ Department of Microbiology PathWest Laboratory Medicine WA, QEII Medical Centre Nedlands Western Australia Australia; ^10^ School of Medicine, University of Western Australia Perth Western Australia Australia; ^11^ Department of Infectious Diseases Perth Children's Hospital Nedlands Western Australia Australia; ^12^ Child and Adolescent Health Service Perth Western Australia Australia; ^13^ WHO Collaborating Centre for Reference and Research on Influenza, VIDRL, Doherty Institute Melbourne Victoria Australia; ^14^ Department of Microbiology and Immunology University of Melbourne Melbourne Victoria Australia

**Keywords:** disease burden, respiratory syncytial virus (RSV), severe acute respiratory infection (SARI)

## Abstract

**Aims:**

Primary aim was to review severe acute respiratory infections (SARI) hospitalisations caused by respiratory syncytial virus (RSV) in children aged < 2 years in paediatric hospitals in Australia. Secondary aims included RSV subtyping, assessing RSV seasonality and contributing to the World Health Organisation's RSV surveillance programme.

**Methods:**

We prospectively reviewed the medical records of children (< 2 years of age) with a confirmed SARI who were admitted to one of four major Australian paediatric hospitals and had a respiratory sample analysed by Polymerase Chain Reaction (PCR). A detailed dataset was completed for RSV positive cases.

**Results:**

Between 1 January 2021 and 31 December 2022, 2290 RSV (laboratory‐confirmed) admissions were identified (53.4% of all SARI admissions). Approximately 50% of all RSV cases were aged 0–6 months. RSV‐A predominated in 2021 with peak infections observed in summer while in 2022 RSV‐B predominated with peak infections in the more traditional winter months.

The median total length of stay (LOS) for RSV positive admissions was 46 h (IQR: 22–82 h). 9% of these children required an ICU admission with a prolonged median LOS 68 h (IQR: 40–112 h). Respiratory support utilisation was consistent over the 2 years. 1.8% required mechanical ventilation; 4.6% continuous positive airway pressure; 23.3% high flow oxygen; and 50.8% low flow oxygen.

**Conclusions:**

RSV in children continues to cause a significant disease burden at Australian tertiary paediatric centres. Ongoing hospital surveillance is required to document the impact of RSV preventative therapies that have become available in 2024.

**Summary**

- What is already known on this topic?
  ○ RSV is a major contributor to respiratory infection hospital admissions in young children, especially in those < 6 months of age.
  ○ The seasonality of RSV was impacted by the COVID‐19 pandemic.
- What this paper adds?
  ○ A multi‐jurisdictional analysis on the impact of RSV on the Australian paediatric healthcare system including treatments, length of stay, and medical interventions utilised.
  ○ Geographical comparison of cases from four tertiary paediatric hospitals.
  ○ Provides a baseline for comparison following the introduction of RSV preventative therapies in Australia from 2024 onwards.

## 1 Introduction

Respiratory syncytial virus (RSV) is a common childhood infection, presenting clinically most frequently as ‘bronchiolitis’, a severe acute respiratory infection (SARI). Global annual episodes in infants aged 0–6 months were estimated to be 1.4 million RSV‐associated acute lower respiratory infection hospital admissions (1.0–2.0 million) [1]. Australian RSV epidemiology also confirms the highest RSV disease burden is in infants and children < 2 years of age [2]. However, Australia's surveillance data collection does not currently uniformly capture information about the incidence of SARI amongst children, nor does it capture clinical details on their management, length of stay (LOS), nor the broader health services impact.

As a result of the COVID‐19 pandemic restrictions, RSV circulation was significantly reduced both nationally and internationally. With the removal of these restrictions in Australia in 2021 and 2022, circulation rebounded back to pre‐pandemic levels [3, 4]. Palivizumab, a short‐acting monoclonal antibody (mAb), was the main RSV prophylaxis available at each hospital. This is a high‐cost, five‐dose scheduled drug so had limited use. Palivizumab was restricted to only the most high‐risk children—generally only neonates born ≤ 28 weeks gestation with uncorrected congenital heart disease, chronic lung disease on oxygen or being of First Nation background.

Recently a number of RSV preventive therapies have been approved by international regulatory authorities, including in the USA and Europe [5, 6]. In 2024, three were approved by the Australia Therapeutic Goods Administration (TGA): Beyfortus (Sanofi), ABRYSVO (Pfizer), and AREXVY (GSK).

Beyfortus is a long‐acting mAb (Nirsevimab) targeted at neonates and infants born in or entering the RSV season and children < 2 years of age at increased risk of severe RSV disease [7, 8]. Preliminary data for the mAb from Spain and the United States has shown promising results in preventing infants being hospitalised with RSV during the northern hemisphere 2023–2024 season [9, 10, 11, 12]. Recently the states of Western Australia, Queensland, and New South Wales announced variable RSV Nirsevimab programmes for infants commencing in April 2024 [13, 14, 15].

ABRYSVO is a maternal RSV vaccine designed to provide protection to the infant via in utero antibody transfer [16, 17]. Abrysvo maternal RSV vaccine became available on the private maret in 2024, and is going onto the National Immunisaiton Program (NIP) in 2025. It has also been approved by the TGA for adults 60 years and over.

Both the vaccine and the long‐acting mAb products can provide RSV protection in children for up to 6 months.

AREXVY is a recombinant RSV vaccine targeted at adults aged 60 years and older. It was approved by the TGA on 16 January 2024 [18] and is currently available via the private market in Australia. The Australian Technical Advisory Group on Immunisation (ATAGI) has provided clinical guidance outlining the priority groups for this preventative therapy [19]. Monitoring the use of antivirals was out of the scope of this study.

Ongoing, robust hospital surveillance, complimented by targeted community surveillance, will be crucial to determine the impact of these and future RSV preventatives therapies and assisting in their evaluation at both an individual and societal level.

## 2 Materials and Methods

**FIGURE 1:**
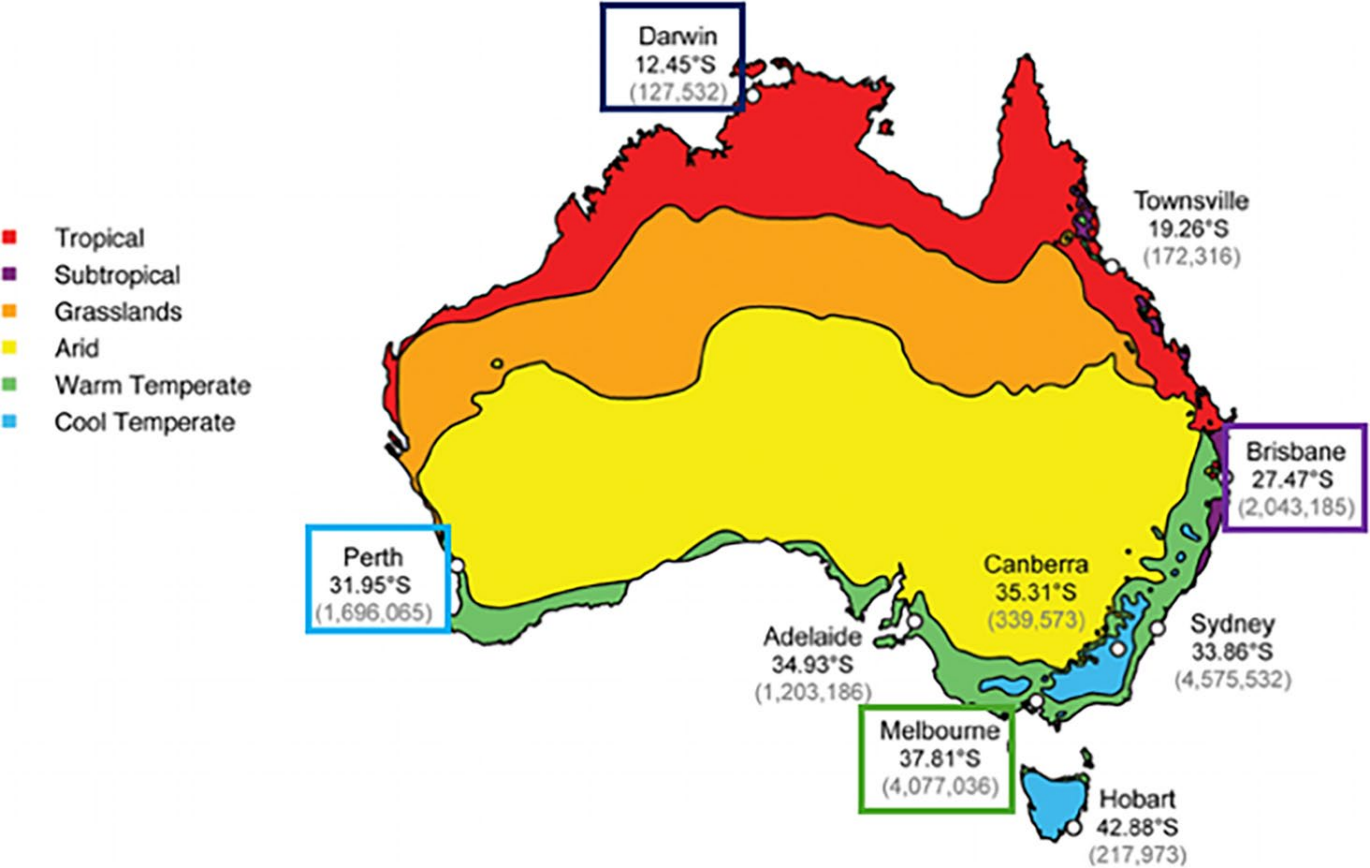
Surveillance sites.

Four large tertiary paediatric hospitals/centres in diverse geographic and climatic areas with varied socioeconomic populations were surveillance sites: the Royal Children's Hospital (RCH), Melbourne, Victoria (Vic); Royal Darwin Hospital (RDH), Darwin, Northern Territory (NT); Queensland Children's Hospital (QCH), Brisbane, Queensland (QLD); and Perth Children's Hospital (PCH), Perth, Western Australia (WA) (Figure 1). These hospitals are the major paediatric hospitals in their jurisdiction but also take patients from neighbouring Australian regions. Some, especially the RDH, also accept patients from the broader Asia‐Pacific region and have a higher percentage of patients of Aboriginal and Torres Strait Islander origin. Cross‐site ethics approval with a waiver of consent was granted by The Royal Children's Hospital Human Research Ethics Committee (HREC 37185). RDH obtained separate approval from the NT Health and Menzies School of Health Research (2017‐2775).

During 2021 and 2022 local researchers screened the hospital admissions and/or laboratory issued Polymerase Chain Reaction (PCR) respiratory assay reports on a regular basis for patients tested for RSV. The hospital records of tested and admitted patients only were screened to determine if they met the inclusion criteria, irrespective of their diagnosis, ICD10 coding or RSV status.

### 2.1 Inclusion Criteria

1. Hospital *admitted* patients aged under 2 years who had a conclusive RSV diagnostic PCR during the study period and met the WHO extended SARI definition [20].
*S*evere—requiring hospitalisation;
*A*cute—presentation within 10 days of symptom onset.
*R*espiratory *I*nfection—clinical presentation of cough or shortness of breath.
  In children < 6 months, apnoea was also included.

Note that sepsis which is also part of the official WHO extended definition was not included due to the common clinical practice of immediately treating and labelling severe illnesses as ‘suspected sepsis’. Fever is not part of the extended SARI definition.

2 Tested positive to RSV (case) or tested negative to RSV (control).

Children who were tested in the emergency department (ED) or outpatient clinics without subsequent admission to a ward were excluded. In 2021, a hospital admission was defined as a stay in any ward including ED short‐stay ward for > 4 h. In 2022, the definition was modified to exclude admissions to the ED short‐stay ward. The ED short stay ward is separate from the core ED to which presentation has never been considered an admission.

### 2.2 Data Collection

Detailed demographics, clinical signs at presentation, and risk factors for severe RSV disease, were collected as per a standardised case report form (CRF), via medical record review of eligible RSV positive cases. A minimal dataset was collected for RSV negative cases. Due to the large number of PCR performed at PCH and the use of paper‐based hospital records, they reported on RSV positive cases and a subset of RSV negative controls. This subset included those testing positive to a pathogen on the panel other than rhinovirus/enterovirus, which is also what other sites focused on.

### 2.3 Laboratory Procedures

Clinical respiratory sample swabs were collected at each hospital and processed in‐house by real time PCR. RSV typing was conducted in the local hospital laboratory or subsets were sent to the WHO Collaborating Centre for Reference and Research on Influenza (WHOCC).

#### 2.3.1 Respiratory Support

Respiratory support utilisation for RSV SARI cases was categorised as either mechanical ventilation (MV), continuous positive airway pressure (CPAP), high flow oxygen (HFNP), or low flow (LF) oxygen via nasal prongs or other methods.

## 3 Results

### 3.1 Case Numbers and Seasonality

Data were collected on 4292 SARI presentations over the 24‐month study period. 54% of these were RSV positive (Table 1).

**TABLE 1:** Snapshot of RSV SARI key results for the 2‐year period.

	RSV pos	Total SARI	% RSV pos
RDH	211	474	45
QCH	503	1390	36
RCH^a^	743	1390 (2048)	53 (36)
PCH^b^	833	1038 (2301)	80 (36)
PICU direct	161	338	48
Gender: Male	1279	2489	51
Aboriginal and/or Torres Strait Islander^c^	230	465	49
Median age in months (IQR)	6 (2–13)	7 (2–14)	

^a^ RCH had an additional 107 and 551 confirmed negative SARI cases in 2021 and 2022, respectively which did not have a CRF completed. These numbers are included in parenthesis.

^b^ PCH estimate they had a total of 451 negative SARI cases in 2021 and 1017 in 2022. These numbers are included in parenthesis.

^c^ Status not known in 19 of the total SARI population reported on.

**FIGURE 2:**
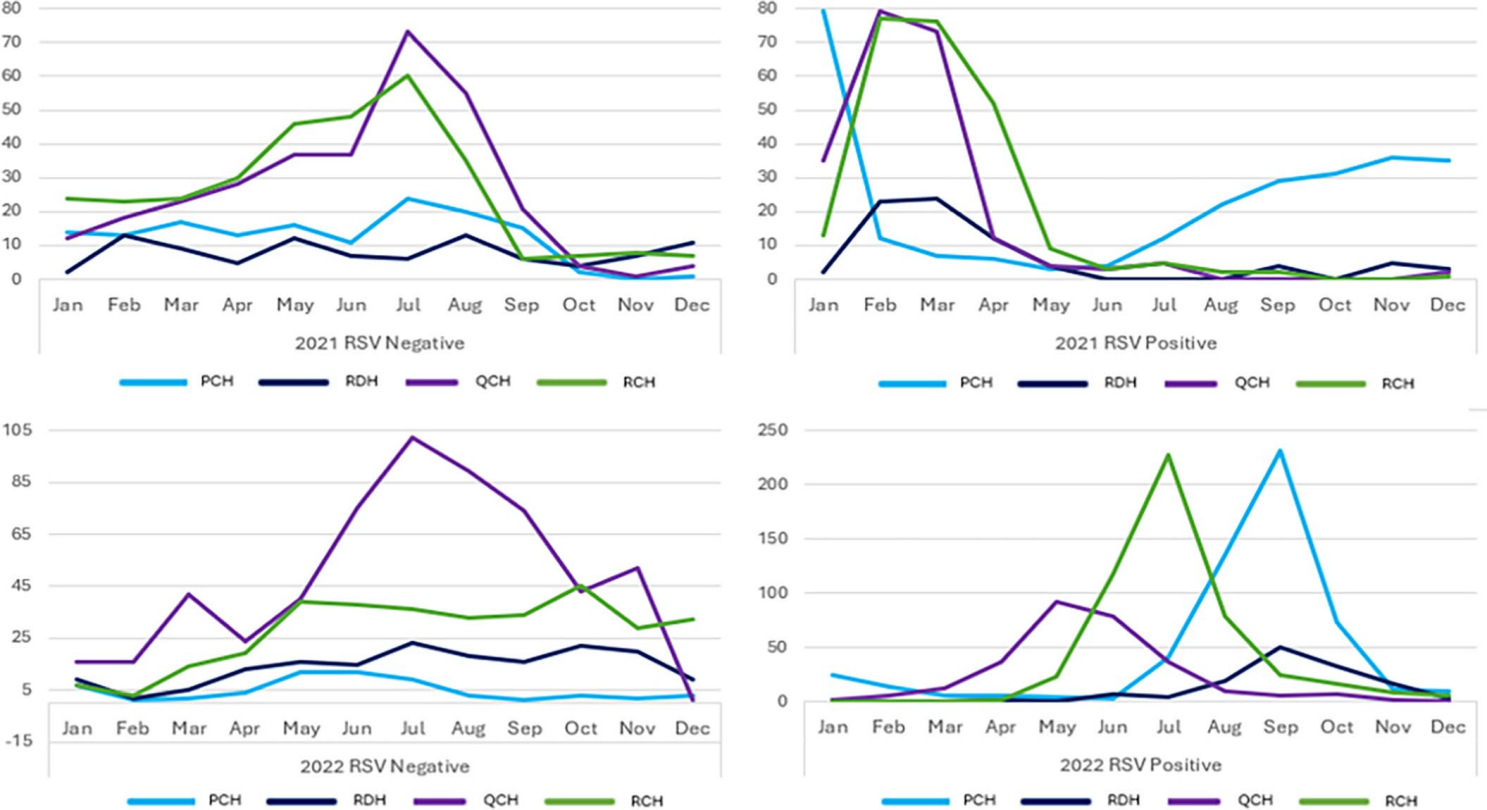
Seasonal distribution of the number of RSV positive and negative SARI cases reported on across each site and year. Note that RSV peaked in Western Australia in late 2020, rather than in early 2021 and their levels rose again post June 2021. Only QCH and RDH completed CRFs on all RSV negative SARI cases in both years.

In 2021, RSV positive SARI cases peaked during the summer months of January and February (RCH, QCH, PCH) in contrast to the traditional autumn‐winter season (May to July) (see Figure 2). RDH had a standard seasonality pattern, with a peak during the rainy season (February–March). In 2022, RCH, QCH, and PCH saw RSV return to a more normal winter pattern but still with some variations: QCH peaked in May, RCH in July, and PCH in September. RDH unusually peaked in September (dry season).

In 2021, the RSV negative SARI cases peaked in July and August at RCH and QLD, much later than the RSV positive SARI cases. There was no distinct peak in RDH and PCH. In 2022, only QCH showed a distinct non‐RSV peak in July, 2 months later than the RSV peak. The other sites showed a spread of RSV negative cases across the year.

### 3.2 Demographics

**FIGURE 3:**
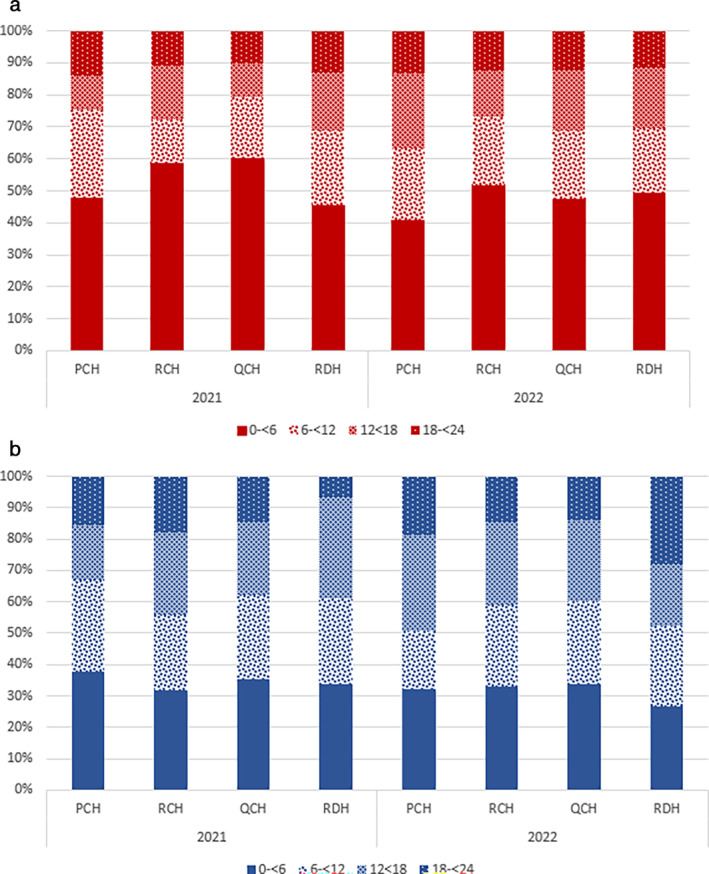
(a) Percentage of RSV positive cases per age group per site. (b) Percentage of RSV negative cases per age group per site.

Overall, more males were hospitalised with a SARI than females regardless of their RSV status (58%) (Table 1). Age distribution varied according to RSV status, reporting year, and study site (Figure 3a,b). Around 50% of all RSV positive cases occurred in the 0 – < 6 month age group, compared with 33% of other‐cause SARI. Most sites saw yearly variations in the other age groups. For example, non‐RSV infections in the 18 – < 24 month age range peaked in 2021 at the RDH (Northern Territory), and in 2022 at PCH (Western Australia).

**FIGURE 4:**
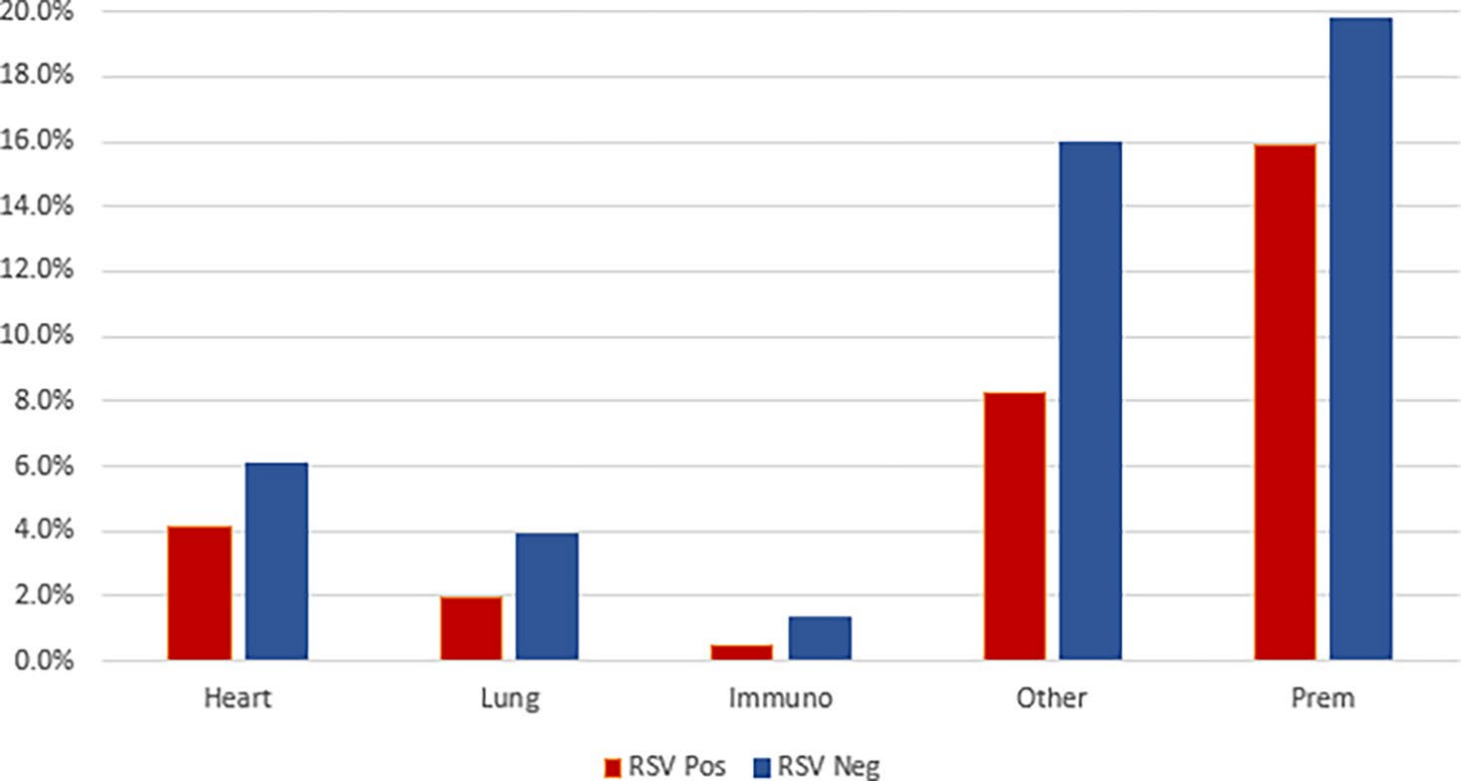
Percentage of SARI cases with known comorbidities for the 2‐year reporting period. Prematurity is classed as < 37 weeks gestation. Note that the gestational age was unknown in 3.8% of negative and 3.2% positive cases. Children may have had more than one comorbidity. Heart, cardiac disease/abnormality; lung, respiratory disease/abnormality; immuno, immunocompromised for any reason; other, any other chronic comorbidity.

SARI patients with congenital heart disease, chronic lung disease, being immunocompromised, born premature, or with other risk factors did not have a higher proportion of confirmed RSV compared with patients without risk factors (Figure 4).

### 3.3 RSV Typing

**FIGURE 5:**
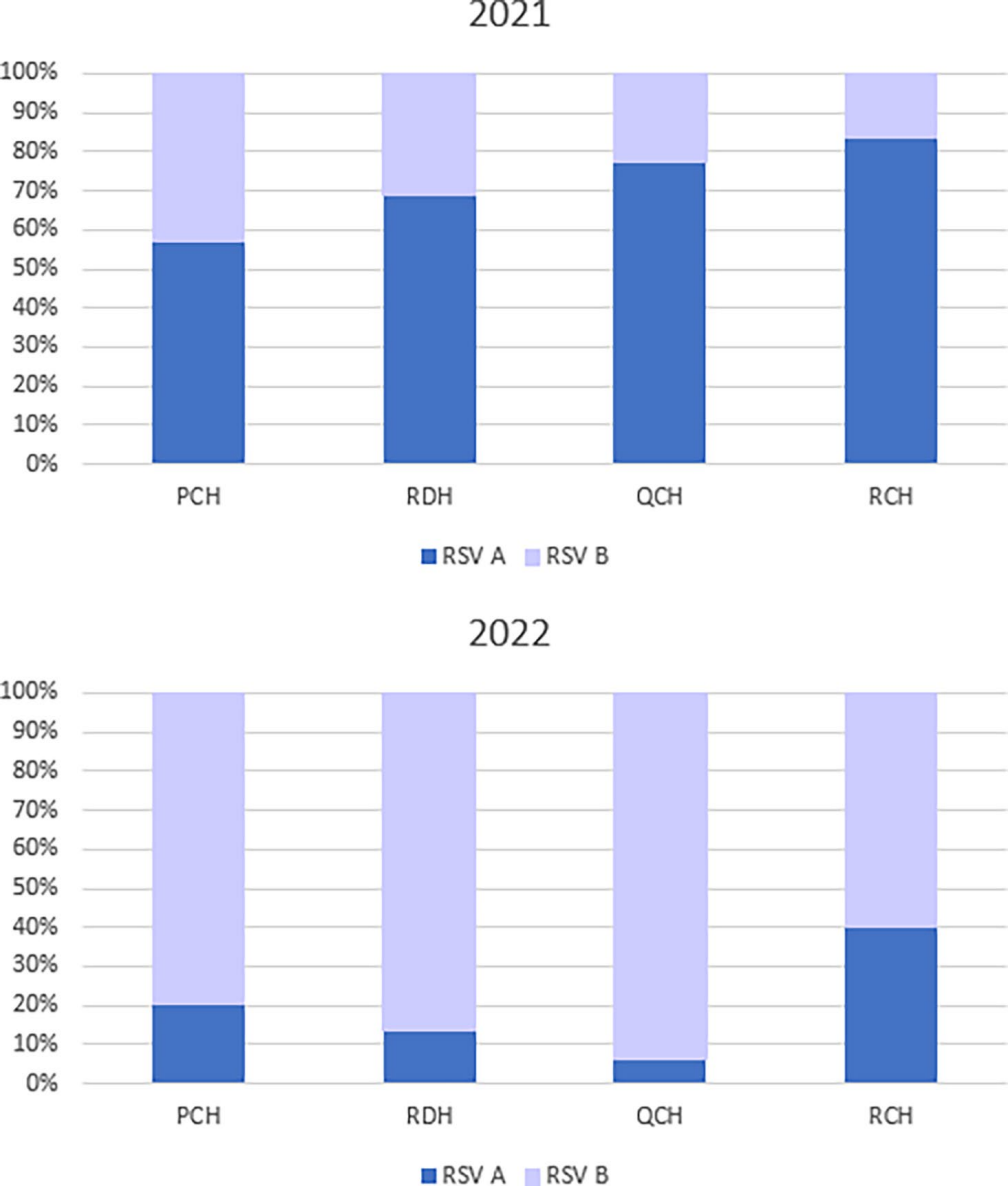
RSV subtype proportions by jurisdiction and year [2021–2022].

Only certain full‐panel PCR tests allow typing of RSV. In this study 556 (69.9%) samples were typed for RSV‐A, RSV‐B, or mixed RSV‐A/B in 2021 along with 790 (53.6%) in 2022. RSV typing showed a marked difference in prevailing RSV types between the 2 years (see Figure 5). In 2021, 75% were RSV‐A, whereas in 2022, 73% were RSV‐B. RSV‐A/B co‐detections represented a very small proportion of cases in both years (1.3%–1.8%). Similar trends in RSV types were seen across the states over this 2‐year period.

### 3.4 Hospitalisation Characteristics

RSV positive SARI patients utilised 6762 hospital days across all sites and years. Their median LOS was 46 h (IQR: 22–82 h).

#### 3.4.1 ICU Presentations

**FIGURE 6:**
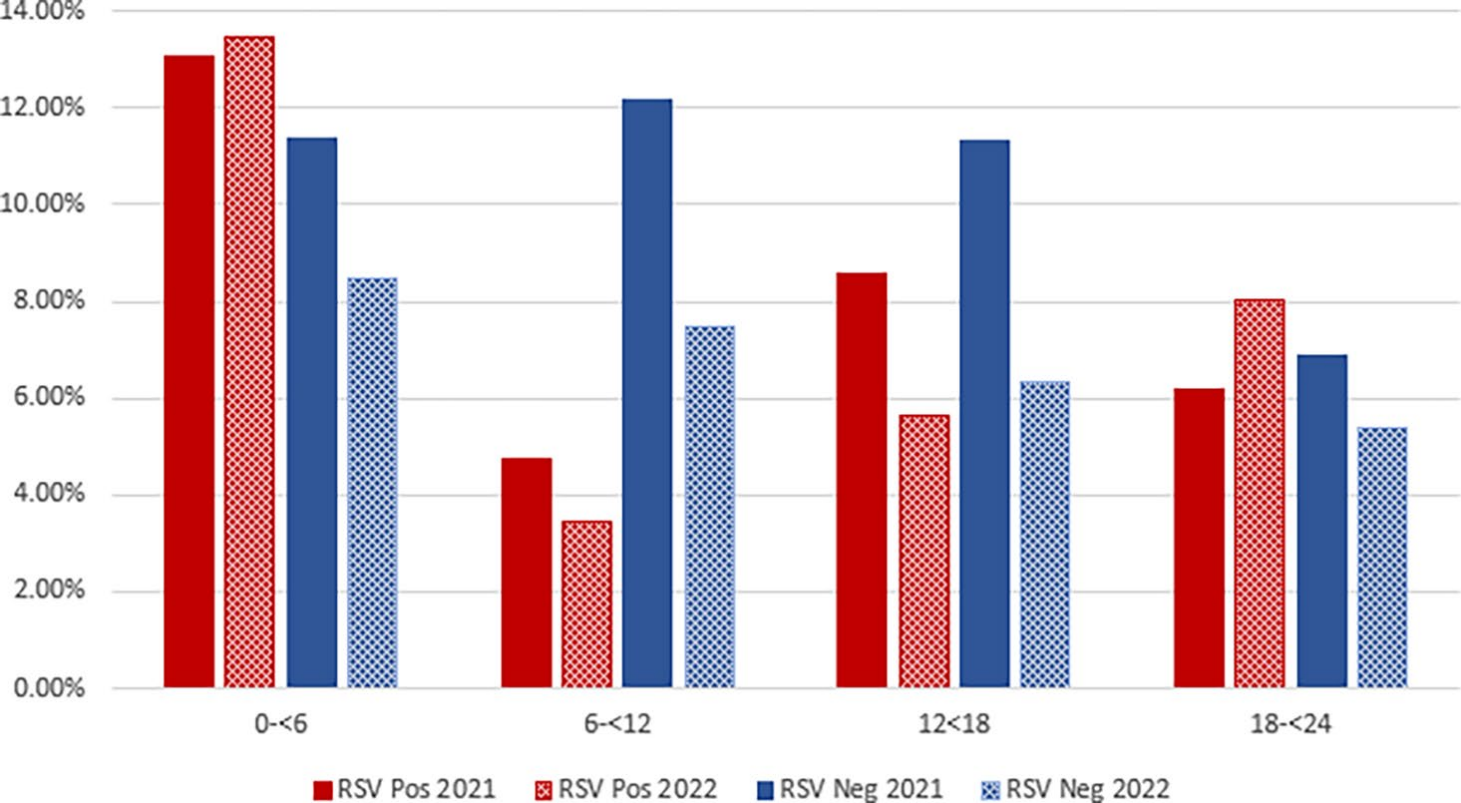
Percentage of total cases admitted directly to ICU over the 2‐year period based on age and infection status.

176 (8.8%) RSV negatives were admitted directly to ICU compared with 162 (7.1%) of RSV positives (Table 1). The youngest cohort (0‐6 m) had the most direct admissions regardless of their RSV status (Figure 6).

**FIGURE 7:**
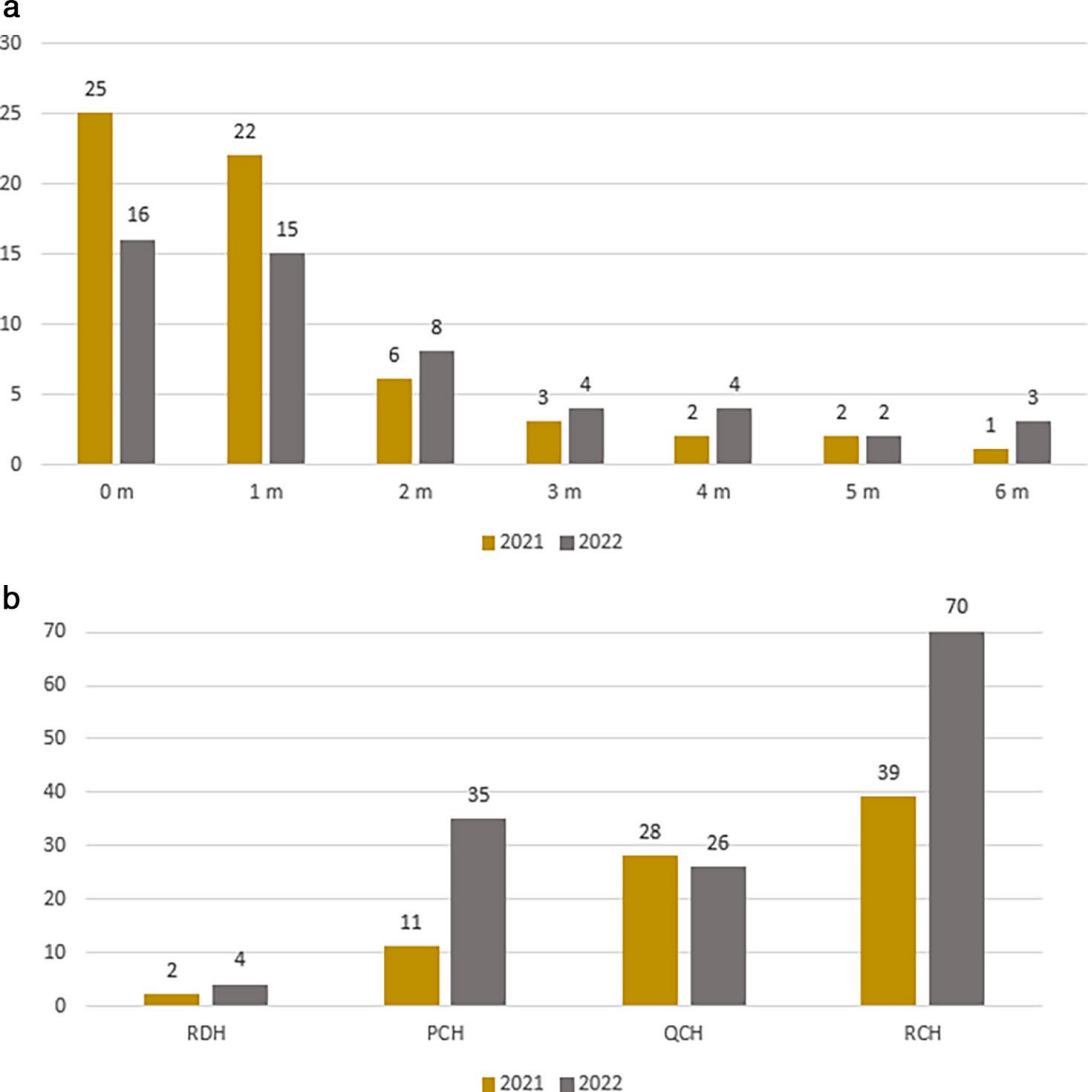
(a) Number of RSV positive cases spending anytime in ICU per age group in those < 7 months. (b) Number of RSV positive ICU admissions at any stage by jurisdiction and year.

Reviewing all ICU stays (direct and later admission) for RSV positive cases, the highest incidence occurred in the 0–1 month age group (Figure 7a). Overall, all sites except for QCH, had more ICU stays in 2022 than in 2021 (Figure 7b). The median LOS was 68 h (IQR: 40–112 h).

#### 3.4.2 Respiratory Support

Respiratory support utilised for RSV SARI cases was categorised as MV, CPAP, HFNP, or LF oxygen via nasal prongs or other methods. Those RSV SARI patients who required oxygen support often received multiple forms throughout their stay, in a step‐up, then step‐down process. Low flow was the most utilised intervention given to 50.8% of the total RSV cohort studied. This was followed by 23.4% on HF, 4.6% on CPAP, and 1.8% on MV. MV is only given in ICU and was required by 18.5% of RSV patients in ICU.

## 4 Discussion

RSV paediatric hospitalisation surveillance is important, detailing the impact of severe infections in children < 2 years post the COVID‐19 lockdowns. Capturing cases across Australia's wide geographical and climatic variability, we identified over 2000 RSV admissions in 24 months from four major Australian paediatric centres, of which 50% were in infants under 6 months. Almost 70% of all RSV ICU admissions were in the 0 – < 2 months age group.

Monitoring the seasonal differences in the occurrence of RSV in SARI cases across Australia to detect early or out of season peaks can help in clinical decision making on when to start at risk children on preventatives such as RSV mAb. It can also potentially guide optimal RSV‐vaccination timing for pregnant women.

It is already known that RSV peaks are more common in winter in the Eastern and Southern states of Australia and varied in Northern WA and NT due to the different climactic regions [21, 22]. Prior to this study however, it was not known if all regions followed similar seasonal and type shifts. We showed that all surveillance sites, except RDH, had unseasonal peaks in 2021 and larger, more in‐season peaks in 2022. Much of these changes in seasonality can be attributed to reopening of many Australian state borders and schools and the lifting other COVID‐19 pandemic restrictions by late 2020. International borders closures with Australia were not fully lifted until late February 2022.

RSV is now a nationally notifiable condition in Australia under the National Notifiable Disease Surveillance System (NNDSS) [23]. It records laboratory confirmed cases (since 2022), but does not capture the clinical impact of RSV, as outlined in this hospital‐based surveillance study.

The cost of paediatric RSV hospitalisations is also substantial, but not easily calculated. Ongoing collection of this hospital surveillance data could provide accurate evaluation of the true cost burden as had been done previously by RCH [24].

### 4.1 Limitations

The authors acknowledge that the limitations of this study include it not being Australia‐wide; not having baseline denominator population data; the reliance of some hospitals on paper records thus reducing reporting capacity; the capacity to make a direct comparison between 2021 and 2022, due to a change in a hospital admission definition; not reporting on all RSV negative SARI cases.

The authors believe that the sites for this project, chosen provide a wide socioeconomic, geographic, and climatic variability for relevant RSV surveillance and do not make assumptions about the rest of the country's SARI incidence. It is not possible to obtain an accurate baseline denominator due to the large and varied catchments of each hospital and due to the very high number of SARI presentations, especially in 2022; it was not possible with the resources available, for all hospitals to report detailed data on all RSV negative SARI cases.

The rationale for excluding the ED short stay admissions in 2022, was because it is predominantly used as a holding bay for patients awaiting test results and not those requiring any medical treatment or intervention. It was not possible to retrospectively exclude the 2021 ED Short Stay cases since this information was not recorded.

The variability in RSV typing was also limited by the site's capacity and funding. With the reduction of typing in 2022, we could not make inferences on impact of RSV type on ICU admissions throughout the study period. In addition, LOS data were not available for all non‐RSV SARI cases, so a comparison using this parameter could not be undertaken.

Future surveillance activities should aim to type the majority of RSV samples and ongoing research is required to determine if RSV type influences disease severity. Finally, the project did not capture special risk group patients (e.g., congenital heart disease) who may have been offered monthly RSV mAb prophylaxis (Palivizumab) at their respective hospitals. As discussed earlier, this is likely to be a very small numbers with two sites estimating it to be < 20 patients per year. The use of all RSV prophylaxis options will be included in ongoing surveillance activities [25].

## 5 Conclusion

With the rollout of new RSV preventative therapies globally there is an increased requirement for robust hospital surveillance to ensure background rates are obtained along with a more comprehensive understanding of the disease burden of RSV. Phase IV data regarding both vaccine effectiveness and safety, as well as ongoing RSV hospitalisation surveillance, will help inform decision‐making regarding all RSV preventative therapies, with the aim to reduce the significant burden RSV has on young children and their families. There will also be a direct comparison between Western Australia (PCH), Queensland (QCH), and other states as well as other international studies, given they are commencing a state‐based long acting mAb (Nirsevimab) programme in 2024. Whilst the NNDS system now records the laboratory‐confirmed RSV case numbers, we believe that this study supports the need for ongoing RSV hospitalisation surveillance at sentinel sites, as its more granular detail is complementary, and paints a picture of the disease burden at the severe end of the RSV clinical spectrum and can also capture some of the direct costs of SARI and RSV hospitalizations.

## Ethics Statement

Cross‐site ethics approval was granted by The Royal Children's Hospital Human Research Ethics Committee (RCH HREC 37185). RDH obtained separate approval from the NT Health and Menzies School of Health Research (2017‐2775).

## Consent

This study was conducted under a waiver of consent per the National Statement on Ethical Conduct in Human Research guidelines approved by RCH HREC.

## Conflicts of Interest

The authors declare no conflicts of interest.

## Data Availability

Raw data were generated at the four hospital sites [RCH, PCH, RDH, and QCH]. Derived data supporting the findings of this study are available from the corresponding author and the lead investigators at each site on request.
